# Ethical bioprospecting and microbial assessments for sustainable solutions to the AMR crisis

**DOI:** 10.1002/iub.2931

**Published:** 2024-12-24

**Authors:** Kayla Cartledge, Francesca L. Short, Alex Hall, Karen Lambert, Michael J. McDonald, Trevor Lithgow

**Affiliations:** ^1^ Centre to Impact AMR Monash University Melbourne Australia; ^2^ Infection Program, Biomedicine Discovery Institute, Department of Microbiology Monash University Melbourne Australia; ^3^ School of Curriculum, Teaching and Inclusive Education Monash University Melbourne Australia; ^4^ School of Biological Sciences Monash University Melbourne Australia

**Keywords:** antimicrobial resistance, ethical bioprospecting, microbial diversity, MRSA, phage therapy, traditional knowledge

## Abstract

Antimicrobial resistance (AMR) has been declared one of the top 10 global public health challenges of our age by the World Health Organization, and the World Bank describes AMR as a crisis affecting the finance, health, and agriculture sectors and a major threat to the attainment of Sustainable Development Goals. But what is AMR? It is a phenotype that evolves in microbes exposed to antimicrobial molecules and causes dangerous infections. This suggests that scientists and healthcare workers should be on the frontline in the search for sustainable solutions to AMR. Yet AMR is also a societal problem to be understood by everyone. This review aims to explore the need to address the problem of AMR through a coherent, international strategy with buy‐in from all sectors of society. As reviewed here, the sustainable solutions to AMR will be driven by better understanding of AMR biology but will require more than this alone to succeed. Some advances on the horizon, such as the use of bacteriophage (phage) to treat AMR infections. However, many of the new technologies and new therapeutics to address AMR require access to biodiversity, where the custodians of that biodiversity—and the traditional knowledge required to access it—are needed as key partners in the scientific, clinical, biotechnological, and international ventures that would treat the problem of AMR and ultimately prevent its further evolution. Many of these advances will be built on microbial assessments to understand the extent of AMR in our environments and bioprospecting to identify microbes that may have beneficial uses. Genuine partnerships for access to this biodiversity and sharing of benefits accrued require a consideration of ethical practice and behavior. Behavior change is needed across all sectors of culturally diverse societies so that rapid deployment of solutions can be implemented for maximum effect against the impacts of AMR.

## 1 INTRODUCTION

**FIGURE 1:**
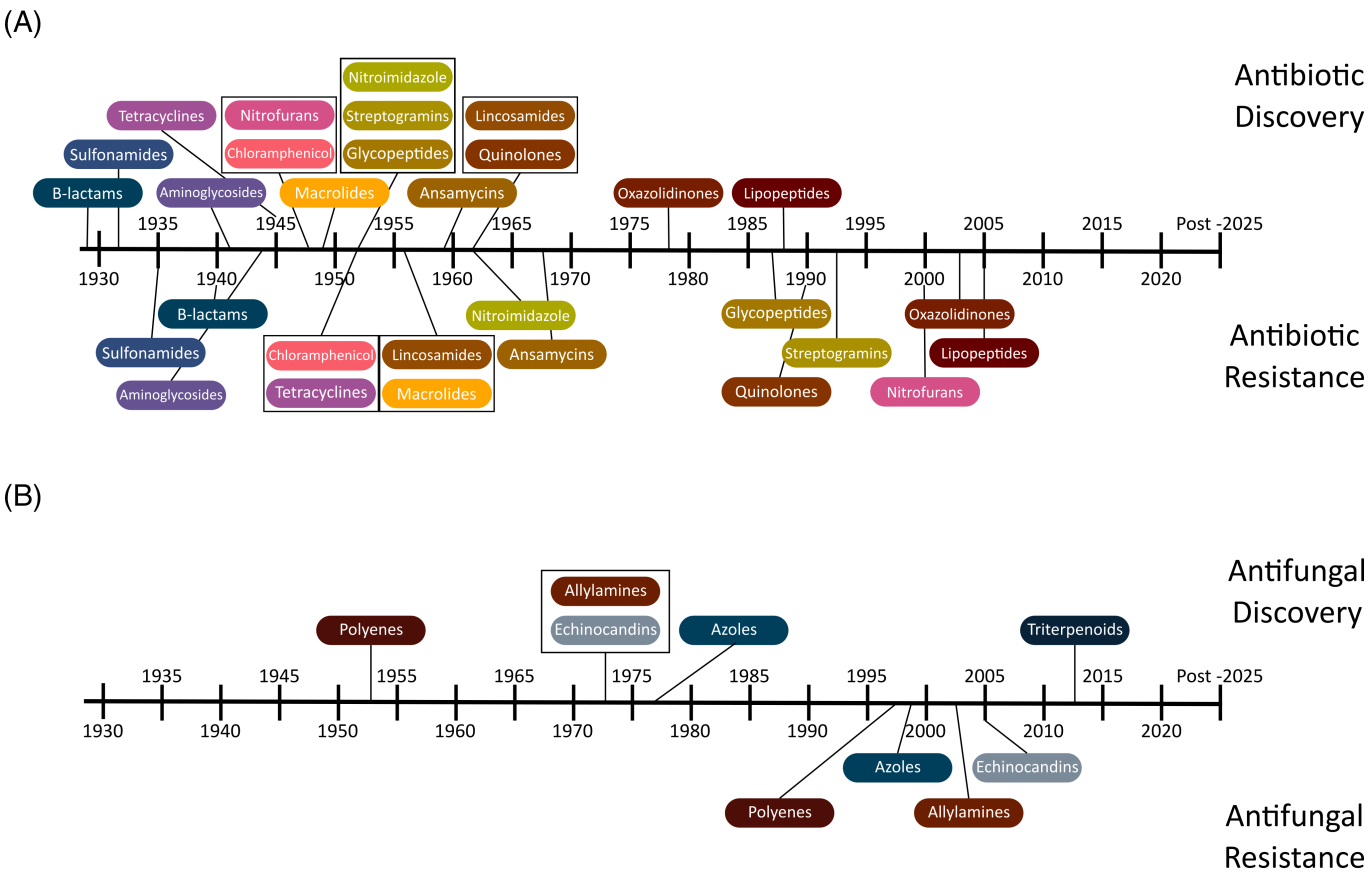
A timeline for the evolution of antimicrobial resistance (AMR). (A) Timeline of antibiotic discovery and clinical deployment (upper labels) and the emergence of substantive antibiotic resistance (lower labels). The 12 dates are drawn from a range of sources. (B) A timeline of anti‐fungal discovery and clinical deployment. The four classes of anti‐fungal drugs are denoted according to the date of a widely known example of that class. Azole resistance is now widespread and other AMR phenotypes are problematic in some places. *Sources*: streptogramin,^1^, ^2^, ^3^ nitrofurans,^4^, ^5^ nitroimidazole,^6^, ^7^ lincosamides,^8^, ^9^ glycopeptides,^10^ oxyazolidinone,^11^ lipopeptides,^12^ quinolones,^13^, ^14^ amsamycins,^15^ chloramphenicol,^16^ tetracycline,^17^ aminoglycosides,^18^ beta‐lactams,^19^ sulfonamides,^20^ triterpenoids,^21^ echinocandins,^22^, ^23^ azoles,^24^, ^25^ and allylamines.^26^, ^27^

“Superbug” is the common‐use name for bacteria and fungi that have evolved and developed antimicrobial resistance (AMR), and in the 20th century, it became dogma that superbugs were something found in hospitals. Overuse of antimicrobial drugs: antibiotic drugs that treat bacterial infections and anti‐fungal drugs that treat fungal infections (Figure 1), by clinicians, was thought to be driving the evolution of AMR strains of bacteria and fungi that would then persist in hospitals and cause hospital‐acquired infections.^28^, ^29^ The 21st century came with surveillance studies that assessed the prevalence of superbugs across cities and non‐urban areas and showed that in most cases people will be exposed to and colonized by superbugs long before they enter a hospital.^30^, ^31^ Establishing causality for the evolution of AMR can be difficult and is anticipated to differ in different locales. In some regions of the world, wastewater from agricultural areas and pharmaceutical manufacturing plants appears to be some of the drivers that inadvertently promote AMR.^32^, ^33^, ^34^, ^35^, ^36^ However, in many places the provenance of AMR characteristics in superbugs remains less clear.

Both the World Health Organization and the World Bank have declared AMR as one of the biggest challenges to public health today and predict it to impact greatly on world economics over the next 10–20 years (Box 1). In this review, we address three points. First, we recognize “AMR” as a difficult terminology that means different things to different audiences. It is critical to work through this difficulty as an “intersectional” approach drawing from professional and public audiences is needed if we are to arrive at sustainable solutions to the problems caused by AMR. Second, that AMR has moved from being considered primarily a clinical issue with clinical solutions to being a community issue with a need for whole community solutions. We explore how current research uses the concept of One Health: addressing agricultural, environmental, and human healthcare together. Third, that since the surveillance of—and some of the solutions to—the AMR problem require work done in the country, Indigenous people, who are the Traditional Owners of that country, need to be genuine partners in that work.

BOX 1 **A summary of the World Bank estimates on projections of AMR.*Health*: Global health costs could rise by up to USD 1000 billion per annum in 2050.**

*Agriculture*: Global livestock production could be reduced by 2.6%–7.5% per annum in 2050.

*Trade*: Global exports could decline by 1.1%–3.8% in 2050.

*Finance*: Global Gross Domestic Product (GDP) could fall by 1.1%–3.8% in 2050. Low‐income countries will be disproportionately affected (with reductions approaching 5% in GDP).

*Poverty*: Growth in extreme poverty could increase by 28.3 million people—again, low‐income countries will be disproportionately affected.

*Source*: World Bank.^37^ and Rupasinghe et al.^38^

## 2 FIRSTLY, *WHAT* IS AMR AND *WHO* NEEDS TO ACT AGAINST IT?

The term AMR means different things to different people, hindering communication. To scientists, AMR is a phenotype that can be used to explain the evolution of superbugs. To clinicians, AMR describes an infection that does not respond to drug treatment. To the general public and policy makers, AMR is a threat to good health, to be mitigated against and avoided. Most considerations of why AMR matters tend to focus on the improvements needed in healthcare/stewardship and drug development in pharmaceutical companies. What is therefore overlooked is the notion that behavior change outside of hospitals is fundamental to implementing solutions to AMR^39^; without changes in community behavior, medical solutions alone are unlikely to prevent the escalation of AMR and the resulting health risks and costs to the world economy.

Other popular discourse on AMR tends to emphasize scientific discoveries and technological developments, and as a result, builds the perception that AMR is primarily a scientific issue with scientific solutions, rather than one where behavior change is needed to implement scientific advances.^40^ There is therefore an urgent need for education of lay audiences, communities, consumers, and individuals. In this task, educators are not starting from scratch and can build on the knowledge and behavior changes established during the COVID‐19 pandemic.

One of the legacies of the COVID‐19 pandemic is that society at large now appreciates the critical role microbes play in our health and environment. The pandemic underscored the importance of community‐focused hygiene, leading to changes in behavior for increased use of soaps and sanitizers, especially those with antimicrobial additives.^41^ Now, post‐COVID, a further change is needed in removing antimicrobial additives from soaps and detergents since these agents promote resistance to all sorts of antimicrobials. Thus, behavior changes focused on clever and appropriate use of hygiene practices are essential in moving against AMR. But this also reveals a second type of behavior change needed, given important socio‐cultural disparities remain even with regard to the uptake and continuance of relatively simple practices such as hand hygiene.^42^, ^43^, ^44^

Responses to the COVID‐19 pandemic exposed gross failures in protecting the most vulnerable in our communities, so we now know that moving effectively against AMR will mean behavior change addressing ageism and racism.^45^, ^46^, ^47^ For example, Italy's experience with 100s of deaths of people in aged care within the first weeks of the pandemic starting serves as a somber reminder of the importance of prioritizing the health and well‐being of the elderly, and yet these actions exposed ageism.^48^ Additionally, across the world, Black, Indigenous, People of Color (BIPOC) communities experienced the pandemic disproportionately to white people due to long‐standing systemic injustices and inequities.^49^, ^50^, ^51^ Even the simplest preventative methods were only available to those with the social capital and support to change their hygiene behaviors; those with the least resources had higher rates of severe illness and mortality.^45^ Recent research suggests that without major behavior change, this will continue to be the trend with AMR in the future.^52^, ^53^, ^54^, ^55^

In a seminal paper, Crenshaw^56^ defined intersectionality as a framework for understanding how various forms of social stratification: race, gender, and class, interconnect and create unique modes of oppression and privilege. Intersectionality is not a comprehensive theory of identity, but a provisional concept that links contemporary politics with postmodern theory.^56^ With regards to good health and improved health outcomes, intersectionality research explores how multiple forms of oppression intersect and shape how marginalized social groups experience health issues.^57^ This same approach can be applied to considering the experiences of Indigenous peoples, who experience the multiple intersecting effects of racist and colonial structures and systems of power. Accordingly, we call for the education of frontline AMR professionals—policymakers, biologists, and clinicians—about intersectionality and anti‐racism across various disciplines, contexts, and institutions. We propose intersectionality as a theoretical lens through which to approach microbial assessments and bioprospecting on traditional lands.

A crucial role of education is to identify target behaviors, raise awareness, develop skills, and adopt health‐enhancing behaviors.^39^, ^40^, ^44^ Education about AMR and the microbes that cause it could occur holistically via curriculum integration across multiple subjects in schools: biology, chemistry, health education, and even language, humanities, and arts. Teacher choice of pedagogies can also aid the learning process. For example, the use of teaching and learning methods: project‐based learning, collaborative learning, role‐playing and simulations, critical thinking and debate, community involvement, alongside the use of technology creates more authentic learning experiences. An interesting example from Spain highlights the use of innovative gamification tools, including card games, online challenges, and social media campaigns, to educate young people about AMR.^58^ These kinds of integrated approaches connect and repeat complex ideas across different contexts. This deepens the understanding of young people and increases the potential for application of their learning to their personal, familial, and cultural lives outside school.

In Australia, a good example of the ways in which schools can directly benefit Indigenous communities and young people is displayed in the Kits‐Mas campaign by DeadlyScience. The Kits‐Mas campaign provided 166 microscopy kits to Indigenous learner classrooms across Australia. The kits included a microscope, reagents, and tools for sample preparation and learning materials. By distributing the microscopy kits to primary schools, the initiative aims to inspire curiosity and promote scientific exploration among Indigenous students. In the United States, the Science‐Education Alliance SEA‐PHAGE and SEA‐GENE programs are directed at informing undergraduate and high school students on microbial assessments and bioprospecting for therapeutic use in the context of infection control and AMR.^59^ Their reports since 2008 show that the Science‐Education Alliance has supported more than 50,000 undergraduate students who spent the time to isolate more than 23,000 phages—viruses that infect and kill bacteria—that have been archived. Of these, more than 4500 have been sequenced to provide a digital sequence identifier in public databases. These phages are currently considered an example of a treatment that can be deployed against infections caused by a superbug where drugs are not effective.^60^, ^61^

## 3 SECONDLY, *WHY* HAS THE PROBLEM OF AMR EVOLVED AND *WHERE* IS IT?

An over‐reliance on the use of too few antimicrobial drugs, failures in the marketplace that made antimicrobial drug development non‐profitable, and a lack of funding to the researchers that would want to focus on new sustainable solutions to the problem of AMR, have all conspired to create a continued and breath‐taking rise in deaths and disability caused by infections that can no longer be treated. The decreased effectiveness of the currently available drugs (Figure 1) means that our options for treating superbugs are significantly reduced.^62^, ^63^ Periods of hospital care are becoming extended and more costly, and AMR‐related deaths are on the rise. The cost to human health and well‐being is huge: there is a consensus use of a figure of around 10 million deaths every year due to AMR infections being a reality by 2050, but we may well be there already, given that in 2019 nearly 5 million AMR‐associated deaths were identified.^64^

**FIGURE 2:**
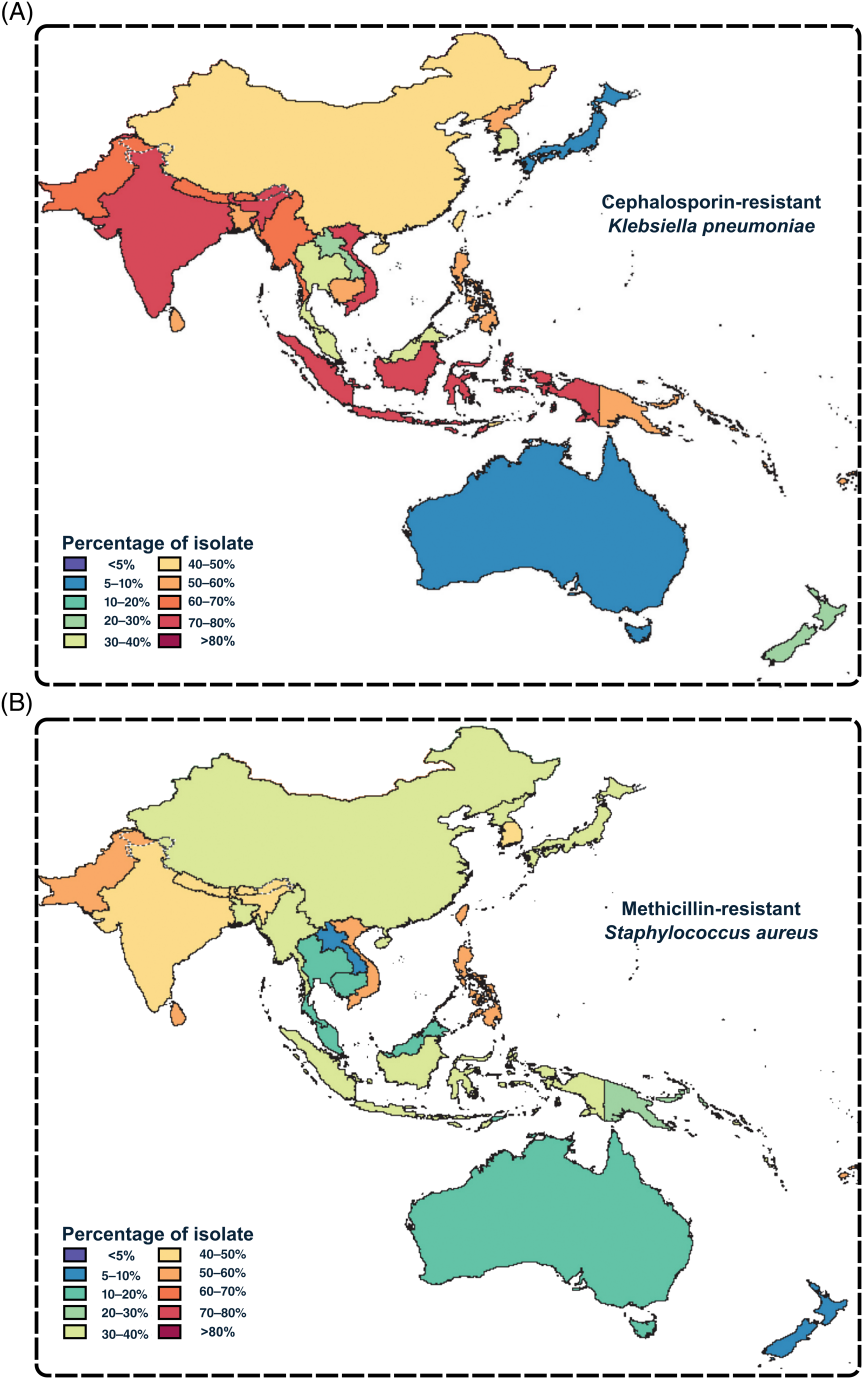
The evolution of antimicrobial resistance (AMR) in the Indo‐Pacific region. The world map redrawn from global 2019 data analyzed by the Antimicrobial Resistance Collaborators.^64^ The Indo‐Pacific region is extracted to show the regional percentage of microbes with AMR, colored for (A) third‐generation cephalosporin‐resistant *Klebsiella pneumoniae* and (B) methicillin‐resistant *Staphylococcus aureus*.

In unpicking these figures and evaluating the scale of the AMR problem, it is important to note that deaths *associated with AMR* are almost always deaths caused by AMR. While many of these deaths have been attributed to an underlying disease (e.g., cancer), the death was caused by an acute superbug infection, such as for cancer patients whose successful surgeries are negated by a fatal infection, women whose successful cesarean surgery is followed by a fatal infection, and cystic fibrosis patients who recover after successful lung transplant but die from a fatal infection. It is because of AMR, because the current drugs could not resolve the superbug infection, that these deaths are counted even though the cause of death may be cited as cancer, childbirth, or cystic fibrosis.^65^ Geographically, AMR is a pervasive problem across all regions of the globe. The authors of this review live in the Indo‐Pacific region of the world (Figure 2), a region with countries least affected by AMR (Australia and New Zealand) as well as countries ranked among those most affected by AMR (e.g., China and India).^64^ It is therefore incumbent upon us to work with partners throughout the Indo‐Pacific region to share knowledge and sustainable solutions across the region. Many of the sustainable solutions to AMR require surveillance work on land and water to provide knowledge for interventions. An equivalent regional view will be required in all parts of the globe.

### 3.1 What is microbial surveillance and how is it implemented?

Microbial surveillance—the assessment of a given region, locale, or building for its resident microbes—has the potential to dramatically improve outcomes in the AMR crisis by providing up‐to‐date distribution and prevalence data on pathogens in order to guide effective interventions to reduce or remove AMR.^66^ The need for comprehensive assessments to generate high‐quality surveillance data on bacterial and fungal superbugs has been identified as a requirement for effective antibiotic action in Australia and globally, as shown by the inclusion of surveillance objectives in WHO frameworks and the national AMR strategy roadmaps that the WHO requests from each country of the world.^67^, ^68^ In the One Health framework, equal weight has been placed on the health of humans, animals, and the environment,^69^ in recognition of the fact that microbial surveillance and assessments show that AMR threats often originate outside of clinical settings.^70^, ^71^

Although the value of the One Health framework is widely recognized, established microbial surveillance systems do not currently meet the goals of this approach.^66^, ^72^ Limitations arise from a lack of microbial surveillance data, particularly in the case of environmental AMR,^73^ and from a lack of integration and coordination across veterinary, agricultural, and medical sectors.^66^, ^72^ Since most surveillance systems report on pathogen and AMR gene prevalence, they fail to provide finer details on the underlying mechanisms of resistance or evolution of AMR pathogens and cannot distinguish between transmission of resistant microbes or transfer of the AMR genes themselves. In poorly resourced settings the above limitations apply and are further compounded by incomplete pathogen and resistance prevalence data from healthcare settings.^72^

**FIGURE 3:**
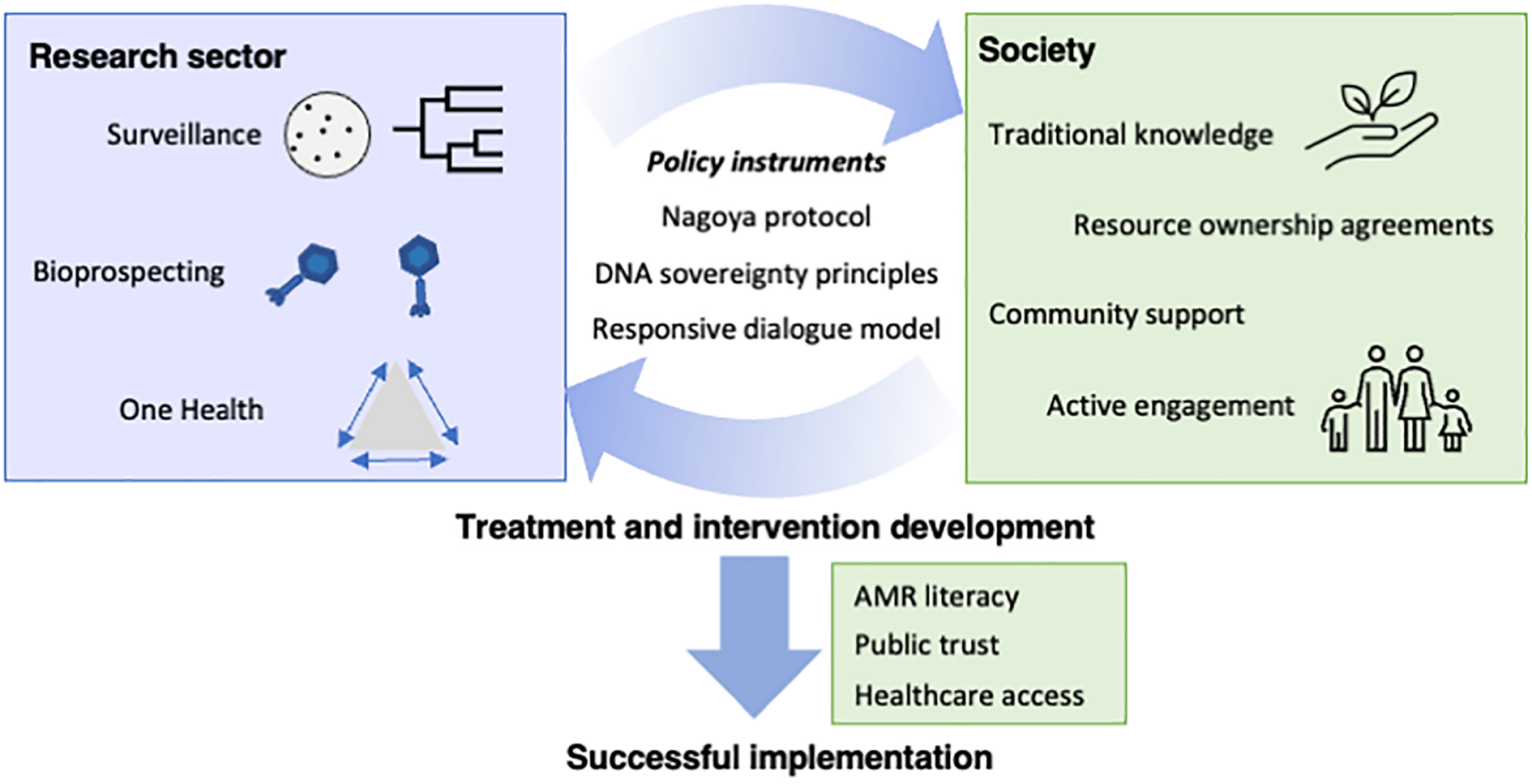
The One Health opportunity for sustainable solutions and interventions to address antimicrobial resistance (AMR). Where previous surveillance enumerates fecal contamination indicator organisms to indirectly indicate infection risk, higher‐resolution information can be obtained from molecular approaches. Examples such as targeted amplification arrays to simultaneously test for multiple pathogens and genomics/metagenomics of environments to monitor pathogens. Successful implementation depends on buy‐in from education (AMR literacy), access to healthcare benefits, and thus an improvement in public trust. This public trust, in turn, can only come through efforts to harmonize all knowledge: traditional, scientific, community‐driven beliefs, converting them toward the actions required for interventions and sustainable solutions to the AMR problem.

A great opportunity exists to move beyond the current low‐resolution and prevalence‐based microbial surveillance. Recent advances have provided means to explore a broader range of questions and interventions, particularly those addressing the environment component of the One Health triad (Figure 3). Traditional surveillance primarily includes enumeration of fecal contamination indicator organisms to indirectly indicate infection risk. Now, higher‐resolution information can be obtained through a variety of genomic or molecular approaches. For example, targeted amplification arrays can directly test for multiple priority pathogens simultaneously, which can aid public health decision‐making in both high‐ and low‐resource settings.^74^ Wastewater‐based epidemiology became popular during the COVID‐19 pandemic as an early indicator of community pathogen spread and can also be applied to AMR genes and pathogens.^75^, ^76^, ^77^, ^78^ Finally, genomics and metagenomics of environmental bacterial isolates and populations can reveal how pathogens evolve and persist in these settings^79^, ^80^; though their cost is beyond what could be met by governments in resource‐poor countries, meaning international investment would be needed.^72^ These approaches would provide a knowledge‐platform to design interventions to minimize environmental pathogen transmission. While deploying and integrating these approaches and leveraging the data generated, will be highly challenging, there remains hope to implement more holistic surveillance in the future that provides public health officials with the level of data needed to develop strong interventions.

## 4 THIRDLY, *HOW* WE WANT TO WORK AGAINST AMR

While *microbial surveillance* is an assessment to see microbes present in a specific environment and how they change over time, a related but distinct activity is *microbial bioprospecting*: discovery of microbes that have scientific interest or commercial value. The ethics of microbial surveillance and bioprospecting differ because surveillance aims to mitigate against costs, while bioprospecting can have the development of a commercial product as an endpoint. Both activities need to consider the Traditional Custodians of the lands and waters that would be included in a comprehensive One Health approach.^81^ Trust needs to be established that sampling will not damage culturally significant environments and resources, that data will be used and communicated in a way that respects the communities concerned, and that Traditional Custodians will be genuine partners in the development of any interventions based on knowledge gained from microbial surveillance and share in any benefits derived from the knowledge gains (Box 2). Partnership to develop interventions is particularly important given that Australian Indigenous people are disproportionately burdened by AMR infections, including from animal or environmental sources.^81^, ^84^

BOX 2 **The Nagoya protocol.**

In 1993 the Convention on Biological Diversity (the Rio Convention) was brought into force, aiming to protect the world's genetic resources. More than 100 countries have ratified this international. The objectives of the Convention on Biological Diversity are “the conservation of biological diversity, the sustainable use of its components, and the fair and equitable sharing of the benefits arising out of the utilization of genetic resources.”

The Nagoya Protocol on Access to Genetic Resources and the Fair and Equitable Sharing of Benefits Arising from their Utilization to the Convention on Biological Diversity (Nagoya Protocol) was adopted on October 29, 2010, as a supplement to the Access and Benefits Sharing provisions in the Convention on Biological Diversity. The Nagoya protocol aims to facilitate the fair and equitable sharing of benefits arising from the utilization of genetic resources for conservation and sustainable use of biodiversity. It also emphasizes the need for genetic resources and traditional knowledge to be shared and used in a fair and equitable way. *Source*: Secretariat of the Convention on Biological Diversity.^82^, ^83^

In Australia, conducting microbial assessments of land and water and bioprospecting for microbes of interest has historically been done without due consideration for the Traditional Custodians of the land and water. While those past and current practices are legal, they are unethical and inferior in quality, as they miss the chance to be informed by important traditional knowledge.^85^ We are working to develop an ethical framework for the collection and surveillance of bacteria, fungi and viruses. Any such framework needs to begin with respectfully acknowledging Aboriginal and Torres Strait Islander peoples as the First Australians and to acknowledge the depth and sophistication of First Nations’ knowledge systems. Without a new respectful and more inclusive approach, 65,000 years’ worth of valuable knowledge grounded in lived experience could be lost and our ability to address problems like AMR made all the harder for it. We suggest that to be effective, the development and implementation of a Framework for Ethical Bioprospecting and Microbial Assessment should be guided by the principles of the Nagoya Protocol on Access and Benefits Sharing (Box 2). The role of microbial surveillance to detect and characterize an AMR pathogen, as well as bioprospecting for phage solutions to treat such a pathogen, is highlighted in the following case study.

### 4.1 Case study *m*ethicillin‐*r*esistant *Staphylococcus aureus*: a common, deadly superbug with prospects for new therapy

One of the first bacteria to be widely publicized as a superbug was MRSA.^29^ Common, community‐acquired infections are caused by the bacterium *Staphylococcus aureus* and, when it evolves to become resistant to the antibiotic methicillin, it is referred to as *m*ethicillin‐*r*esistant *Staphylococcus aureus*, that is, MRSA (Figure 2B). This superbug can also become resistant to other antibiotic classes, including last‐line antibiotics such as linezolid.^86^, ^87^, ^88^ In Australia, a 2017 assessment found that MRSA carriage among Indigenous Australians is disproportionately high, with approximately one‐third of *S. aureus* isolates from skin swabs showing methicillin resistance.^89^ Contemporary microbial surveillance assessments had characterized a sequence type called ST93 as the dominant community‐acquired MRSA lineage,^90^ related to an endemic, widespread, drug‐sensitive ancestor of ST93 that had grown to prevalence across many communities before an epidemic of ST93 MRSA spread through Australia and the Indo‐Pacific region, and then elsewhere in the world.^91^

The skin infections and abscesses in deeper skin layers caused by *S. aureus* are contagious, transmitted through skin‐to‐skin contact, spreading the disease within and between communities. Published evidence shows that children in Indigenous communities suffer skin infections more than children anywhere in the world.^92^, ^93^ Skin infections serve as an entry point for *S. aureus* into the blood causing sepsis^94^ or into bones causing osteomyelitis.^95^ Aboriginal children are disproportionately affected by sepsis (three times more than non‐Aboriginal children) and osteomyelitis (10 times more than for non‐Aboriginal children).^95^, ^96^

As AMR rises, new treatments are sought. As natural predators of bacteria, the viruses referred to as phages are promising treatments for superbug infections, particularly skin infections caused by MRSA that can be treated topically.^97^, ^98^, ^99^, ^100^, ^101^ Phages possess several characteristics that make them ideal for therapeutic use: (i) they display high specificity for their target bacterial species, meaning minimal impact on the human bacterial microbiome, (ii) they have efficacy against infections that are resistant to antibiotics, and (iii) can be easily sourced from the environment, including water sources. Thus, phage therapy represents a medically viable option against MRSA. In many Aboriginal and Torres Strait Islander communities, water sources hold cultural significance and are viewed as places of healing. The presence of phages in these water sources means that phage therapy holds potential to be an acceptable tradition‐based treatment in Aboriginal and Torres Strait Islander communities, due to its consistency with other cultural methods.

Around the world, phage therapy is being trialed for compassionate use cases, where no effective antibiotic therapy is possible.^60^, ^102^, ^103^, ^104^ A recent evaluation of phage therapy outcomes in 35 hospitals in 12 European countries has been published^61^ and MRSA infections were prevalent: of the 100 patients treated, 39 of the cases had MRSA infections of bone or soft tissue, and the study reflected the overall safety of phage therapy across the patients treated. A bacteriophage formulation produced by AmpliPhi Biosciences Corporation consisting of three phages (AB‐SA01) is available for compassionate use. Results with AB‐SA01 suggest killing ~95% of MRSA isolates^105^ and it has been shown to be safe in human trials.^106^ There are several potential advantages in the use of phage therapy for treating MRSA infections^99^, ^100^ and initial work has started on bioprospecting for local phages.^107^ Most phages that have been isolated for therapeutic purposes are isolated from hospital wastewater tanks, on the assumption that the circulating pathogens in that hospital and their attendant phages will be represented in the hospital wastewater.^103^, ^104^ This strategy has proven to work; many useful phages have been isolated by this approach, but a limitation with this sourcing is that only phages currently circulating in that hospital environment will be present in these water samples.

### 4.2 An example of bioprospecting across community environments

**FIGURE 4:**
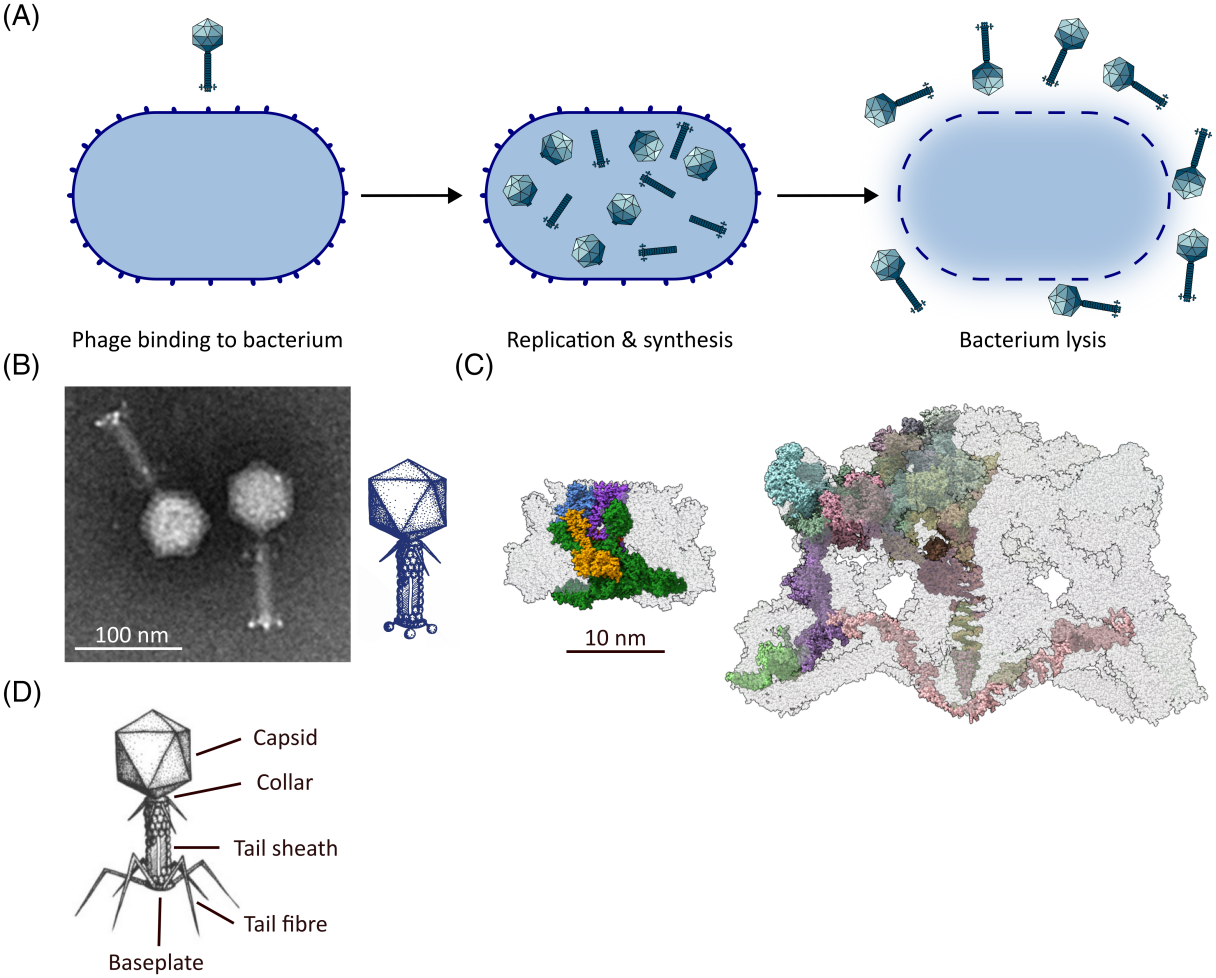
Bioprospecting for bacteriophage that will kill antimicrobial‐resistance bacteria. (A) Summary depiction of phage lysis of bacteria. The initial binding of a phage virion (dark blue) to the cell surface of a bacterium will depend on specific interaction of receptor‐binding proteins in the phage virion—often tail‐fibers or other projections from the base‐plate—to a receptor projecting from the bacterial surface. This then triggers events that result in the replication and synthesis of up to 50 virions, ultimately triggering bacterial cell lysis and death, releasing the phage progeny for future killing of more bacteria. (B) Transmission electron microscopy of phage Merri‐merri‐uth nyilam marra‐natj (MMNM) virions^113^, ^114^ reveals stumpy knob appendages on the baseplate of the phage. Scale bar is 100 nm. (C) Cryo‐electron microscopy of the related phage XM1 (pdb 7KH1; shown in the left panel) with a baseplate composed of five protein subunits as compared to the 13 proteins in the archetypal phage T4 (pdb 5IV7; shown in the right panel). Scale bar is 10 nm. (D) Diagram of MMNM‐like phage from Bunurong country showing the elongate tail‐fibers distinguishing it from phage MMNM.

An alternative approach is bioprospecting for phages in water and soil outside of hospital wastewater tanks by sampling suburban and remote waterways. This strategy provides for a much broader sampling across human communities: phages exist in water and on plants, and therefore in the gut microbiomes of animals, as well as in soils and so in the skin microbiome of animals.^108^, ^109^, ^110^ Diverse phage types have been isolated through culture‐dependent means from freshwater environments, and marine samples ranging from on‐shore to polar ice.^111^, ^112^ Each species of phage has evolved to interact with a single species of bacteria, through a set of carefully choreographed molecular events (Figure 4A) involving binding proteins built into the phage virion that can selectively bind to receptors on the surface of a bacterium.^115^, ^116^, ^117^, ^118^ Correct binding to the surface triggers the release of the double‐stranded DNA genome of the phage across the host cell membranes and cell wall and into the cytoplasm.^115^ This DNA entry reaction is well documented but is as yet poorly understood at the molecular scale. After DNA entry, phage replication ensues to generate up to 100 progeny phage per cell. Accumulation of these phage triggers an explosive lysis of the bacterium, thereby killing it and releasing many phage progeny to go forth and kill more bacteria (Figure 4A). The details of this life cycle mean two important things in terms of how we might use phage to our benefit: (i) the host specificity means that a phage will kill the bacterial species that it is targeted to but will not kill “good” bacteria in a microbiome or microbial community, and (ii) phage numbers are amplified with every cell killed, meaning that great destruction of the host bacterial population can be wrought in just a short time after an application of phage.

In one recent study in partnership with Traditional Custodians of a waterway in Australia, two local phages were isolated as unique test cases to develop a new software tool for characterizing novel phage types.^113^ Found on Wurundjeri country, the phage Merri‐merri‐uth nyilam marra‐natj (abbreviated to MMNM) (Figure 4B) has a minimalist architecture compared to the archetypal contractile phages such as T4.^114^ For example, while phage T4 has a complex baseplate structure composed of 13 proteins to bind to its host bacteria, the minimalized version has only five proteins (Figure 4C). This elegant simplicity promises new insight into how phages recognize their hosts, and new ways of selecting for or engineering phages that can infect new hosts. Furthermore, a related phage isolated from neighboring Bunurong country reveals ways in which species variation can provide a range of new bacterial killing proteins in a set of elegant long tail‐fibers (Figure 4D). In keeping with the principles of the Nagoya Protocol (Box 2), these and other bioprospecting projects use an ethical framework to ensure that access to biodiversity can be made possible and that benefits derived from the work will be shared.

## 5 LASTLY, *WHO* IS MOVING TO ENSURE THAT ASSESSMENTS AND BIOPROSPECTING ARE DONE ETHICALLY?

Calls are being made in regions of the world to access the biodiversity of land and water currently held by Indigenous peoples and Traditional Custodians.^119^ For example, a recent report suggested the importance of phage therapy for Indigenous people in Ecuador, alongside educational campaigns to inform communities about AMR and phage therapy.^120^ One crucial element in ethical bioprospecting is the need for digital sequence identifiers to support the concept of data sovereignty. For example, in the case of bioprospecting, phages isolated from specific lands/water can be recognized by their registered DNA sequence. This DNA sequence information can be registered on the date of discovery, including details of the lands on which it was discovered and the partners who made the discovery, and maintained in confidential form for some time if necessary to the partners who isolated it. This information provides an enduring record of provenance if some future value becomes attributable to the phage. Bioprospecting is, however, a complex situation to navigate in legal and commercial terms.^119^, ^121^, ^122^, ^123^, ^124^, ^125^

Microbes in the Arctic and Antarctic have evolved enzymes and other proteins to reap energy from their frozen environments; these proteins have cold‐adapted properties that make them attractive to biotechnological companies and industrial processes, from the more prosaic (e.g., anti‐freeze proteins) to more creative uses in bioremediation and as pharmaceuticals, so there are substantial commercial drivers to motivate bioprospecting. New Zealand and Australia are key custodians of Antarctica, where science and resources are protected by a strict treaty. The Arctic, on the other hand, is open to exploitation due to the inability of its governing council to make binding laws. This contrast in the governance of the poles is reflected in the differing approaches to bioprospecting in New Zealand and Canada.^126^ Nonetheless, ethical microbial surveillance assessments and bioprospecting in both Canada and New Zealand emphasize environmental stewardship and involve consultation and engagement with Indigenous communities. In New Zealand, the Treaty of Waitangi (1840)—the founding document that outlines a partnership between the British Crown and the Māori—ensures Māori involvement in decisions affecting their lands and resources. The Resource Management Act (1991) incorporates Māori perspectives into environmental planning and mandates that Māori values and traditional knowledge (mātauranga Māori) be considered in resource management.^127^

Canada mandates consultation with Indigenous peoples through the duty to consult, especially when the rights or interests of Indigenous peoples are potentially affected. Canadian researchers conduct microbial surveillance and assessments by culture‐independent (i.e., metagenomics‐based) and culture‐dependent methods for genetic information from microbes in sea ice and in the microbiomes of fish caught by Inuit fishermen at culturally significant sites in Gjoa Haven, Nunuvit. The studies benefit from traditional knowledge (when and where the fish will be and how to catch them under ice). The microbiomes of the fish in turn provide for a health index of the fish themselves and the environment in which they live to be integrated with the knowledge of the Traditional Custodians.^128^, ^129^ This requirement for consultation is stronger and more consistently applied in Canada than in Australia, where provisions for consultation exist, but decisions can be made without the consent of Traditional Custodians. In New Zealand, ethical bioprospecting starts with establishing initial contact with Māori communities (iwi or hapū) and building a respectful relationship. This includes following cultural protocols, clearly explaining the research purpose and methods, and negotiating terms of access and benefit‐sharing. Agreements are formalized in writing, and all activities are carried out in accordance with the agreed terms. This process ensures that Indigenous rights and knowledge are respected, fostering trust and collaboration. However, despite this potential for intersectional work,^130^, ^131^ there are still few studies that result from integration of scientific and Indigenous approaches to research.^132^, ^133^, ^134^, ^135^, ^136^

Are strict laws needed to force researchers to respect Indigenous rights and knowledge? An example to the contrary comes from DNA data sovereignty. Data sovereignty allows a nation or community to control data generated within its jurisdiction, adhering to its laws, governance, and cultural values. In Canada, the government allocated $73 million to the First Nations Information Governance Centre for a national data governance strategy. In New Zealand, partnerships like the Pacific Data Sovereignty Network and Stats NZ support collaborative research with Pacific peoples. DNA data sovereignty ensures Māori control over their DNA data, treating it as a taonga (treasure) under the Treaty of Waitangi, thus protecting its cultural significance. This covers genomic data, biological samples, and DNA from native species. While there are no specific laws proscribing data sovereignty, the combined influence of guidelines, institutional policies, funding requirements, ethics committee reviews, community engagement, professional standards, and international advocacy creates a robust framework that encourages compliance and respect for Māori data sovereignty principles among scientists and universities. The combined influence of guidelines from Te Mana Raraunga, institutional policies from universities, funding requirements from the Health Research Council of New Zealand and the Marsden Fund, ethics committee reviews, community engagement, professional standards advocated by the Royal Society Te Apārangi, and international advocacy from the Global Indigenous Data Alliance creates a robust framework that encourages compliance and respect for Māori data sovereignty principles among scientists and universities.

## 6 CONCLUDING REMARKS

Since the turn of the century, it has become clear that addressing the evolution of AMR and the resultant waves of superbugs that cause infections needs surveillance of community environments: agricultural areas, wild lands, and waterways, as well as regional and remote settlements and built areas of high population density.^71^, ^137^, ^138^, ^139^, ^140^, ^141^ Despite the advances made by adopting One Health strategies in the newest of this work, a major knowledge gap exists in being able to address where and how new AMR threats emerge. This must be addressed if we are to strategically intervene to prevent or reverse these emergent AMR events and to slow its ongoing evolution.

Motivating the behavior change needed across communities depends on tailored educational work, and that should be built into National AMR Action Plans and must be informed by the work of biological research and developments in healthcare. The idea of One Health has served well in conceptualizing plans of action. The need for intersectionality makes the necessary education and behavior change complex, but the discipline‐specific knowledge on AMR and the microbes that cause it is already in hand for this intersectionality to build from.

Understanding the way in which new antimicrobial drugs kill their target microbes, as well as understanding how non‐drug solutions such as phage therapies work, is made possible by the deep knowledge base derived from decades of scientific research. Many of the phages that will be used therapeutically, as well as some of the future drugs, will likely derive from bioprospecting activity. The means by which this bioprospecting can be done ethically, to ensure both access to biodiverse environments and benefit‐sharing, is becoming better appreciated and better publicized. Many of the studies reviewed here show the progress that is being made in countries around the world.

## CONFLICT OF INTEREST STATEMENT

The authors declare that they have no conflicts of interest.
